# Familial wild-type gastrointestinal stromal tumour in association with germline truncating variants in both *SDHA* and *PALB2*

**DOI:** 10.1038/s41431-021-00862-5

**Published:** 2021-04-15

**Authors:** James Whitworth, Ruth T. Casey, Philip S. Smith, Olivier Giger, Jose Ezequiel Martin, Graeme Clark, Jaqueline Cook, Marlee S. Fernando, Phillipe Taniere, NIHR BioResource, Eamonn R. Maher

**Affiliations:** 1 University of Cambridge Department of Medical Genetics, NIHR Cambridge Biomedical Research Centre, and Cancer Research UK Cambridge Centre, Cambridge Biomedical Campus, Cambridge, UK; 2 Department of Pathology, University of Cambridge, Addenbrooke’s Hospital, Cambridge, UK; 3 Department of Clinical Genetics, Northern General Hospital, Sheffield, UK; 4 Department of Pathology, Sheffield Teaching Hospitals NHS Foundation Trust, Royal Hallamshire Hospital, Sheffield, UK; 5 Department of Pathology, University Hospitals Birmingham NHS Foundation Trust, Birmingham, UK

**Keywords:** Cancer genetics, Genetic testing

## Abstract

Gastrointestinal stromal tumour (GIST) is a mesenchymal neoplasm arising in the gastrointestinal tract. A rare subset of GISTs are classified as wild-type GIST (wtGIST) and these are frequently associated with germline variants that affect the function of cancer predisposition genes such as the succinate dehydrogenase subunit genes (*SDHA, SDHB, SDHC, SDHD*) or *NF1*. However, despite this high heritability, familial clustering of wtGIST is extremely rare. Here, we report a mother–son diad who developed wtGIST at age 66 and 34 years, respectively. Comprehensive genetic testing revealed germline truncating variants in both *SDHA* (c.1534C>T (p.Arg512*)) and *PALB2* (c.3113G>A (p.Trp1038*)) in both affected individuals. The mother also developed breast ductal carcinoma in-situ at age 70 years. Immunohistochemistry and molecular analysis of the wtGISTs revealed loss of SDHB expression and loss of the wild-type *SDHA* allele in tumour material. No allele loss was detected at *PALB2* suggesting that wtGIST tumourigenesis was principally driven by succinate dehydrogenase deficiency. However, we speculate that the presence of multilocus inherited neoplasia alleles syndrome (MINAS) in this family might have contributed to the highly unusual occurrence of familial wtGIST. Systematic reporting of tumour risks and phenotypes in individuals with MINAS will facilitate the clinical interpretation of the significance of this diagnosis, which is becoming more frequent as strategies for genetic testing for hereditary cancer becomes more comprehensive.

## Introduction

Gastrointestinal stromal tumour (GIST) is a mesenchymal neoplasm arising in the gastrointestinal tract, most commonly occurring in the stomach or small intestine with around 10% in other locations [1]. DNA sequencing of GIST tissue reveals a somatic driver variant in *KIT* or *PDGFRA* in most cases but in 15% of adult cases and 85% of paediatric instances, no such driver is identified and the tumour is termed a wild-type GIST (wtGIST) [2, 3].

Amongst wtGISTs, more than three-quarters show evidence of succinate dehydrogenase (SDH) enzyme deficiency (known as dSDH-wtGIST), generally detectable by tumour immunostaining that reveals loss of SDHB expression [3, 4]. dSDH-wtGISTs show phenotypic differences to non-wtGIST including multinodular appearance, lymphovascular invasion, and epitheloid/mixed epitheloid spindle cell histology [5] and are less likely to respond to standard targeted therapy (e.g., imatinib) [2, 6]. They are frequently associated with a germline variants affecting the function of the *SDHA*, *SDHB*, *SDHC* or *SDHD* (*SDHX* genes) or somatic *SDHC* promoter hypermethylation. Whereas only 3–4% of non-wtGISTs occur in association with a causative germline variant (e.g. *KIT*, *PDGFRA*), one may be detected in as many as half of individuals with dSDH-wtGIST [3, 4, 7, 8]. The distribution of germline *SDHX* gene variants associated with dSDH-wtGIST differs from that seen in the tumour types most commonly associated with dSDH (phaeochromocytoma and paranglioma (PPGL) and head and neck paraganglioma) in that germline *SDHA* variants are frequent with wtGIST but an infrequent cause of PPGL or head and neck paraganglioma [7, 9]. Despite the high diagnostic yield for causative germline variants in individuals with wtGIST, reports of familial wtGIST are very rare. Whilst two sisters with dSDH-wtGIST and a germline variant affecting *SDHA* function have previously been reported [10], series of 34 (ref. [4]) and 33 (ref. [11]) wtGISTs reported no familial cases.

Here we describe a rare example of familial dSDH-wtGIST where a germline truncating *SDHA* variant was detected. In addition, a germline truncating *PALB2* variant was also found meaning that two cancer predisposition gene variants with implications for genetic counselling were present in the same individual. This situation that has previously been termed multilocus inherited neoplasia alleles syndrome (MINAS). Whilst there is little to indicate that the *PALB2* variant contributed to GIST tumourigenesis, we speculate that this unusual finding of may have led to increased penetrance in this family.

## Materials (subjects) and methods

### Participants

The family was ascertained via a clinical genetics centre after a mother and son presented with wtGIST. Information relating to these individuals has been submitted to the Leiden Open Variant Database and can be found at https://databases.lovd.nl/shared/diseases/04296 (individual IDs 00264003 and 00264004). Both affected individuals gave written informed consent for research investigations and the research was approved by the relevant Research Ethics Committees (South Birmingham REC and East of England—Cambridge South REC).

### SDHB immunohistochemistry

SDHB immunohistochemistry was performed on 3-μm sections of FFPE tissue mounted on adhesive slides. The staining was performed on a fully automated BOND III IHC and ISH stainer system (Leica Biosystems, Nassloch, Germany). The SDHB primary antibody rabbit polyclonal (HPA002868, Sigma Aldrich, St Louis, MO, USA) was used. The Primary antibody binding to tissue sections was visualized using BOND Polymer Refine Detection system (DS9800, Leica Biosystems, Nassloch, Germany).

### Whole-genome sequencing (WGS)

WGS and bioinformatic processing to produce variant call format (VCF) files was performed on samples from study participants as part of, and according to protocols devised by, the NIHR BioResource Rare Diseases study [12]. DNA Libraries were sequenced with an Illumina HiSeq 2500 instrument (Illumina Inc., San Diego, CA, USA). Read alignment to GRCh37 was performed using Illumina Isaac aligner version SAAC00776.15.01.27 [13]. Single nucleotide variants and indels were called from resulting binary compressed sequence alignment map (BAM) files using Illumina Starling software version 2.1.4.2.

### Variant assessment from WGS data

Variants occurring in genes included in a list of 83 cancer predisposition genes were filtered and assessed according to a protocol described previously [14].

### DNA extraction from formalin-fixed paraffin-embedded tumour blocks

Slides were prepared from formalin-fixed paraffin-embedded (FFPE) tumour blocks by the Human Research Tissue Bank, Cambridge University Hospitals. Following de-paraffinisation, slides were reviewed by a pathologist to mark selected tissue and tumour dissection was performed by colleagues in the Department of Haematology and Oncology diagnostic services, Cambridge University Hospitals. DNA was subsequently extracted from resulting tissue.

### AmpliSeq panel sequencing

Library preparation was undertaken in the Stratified Medicine Core Laboratory using a custom AmpliSeq panel (Thermo Fisher Scientific, Waltham, MA, USA) that included the *SDHA* region of interest. An Illumina MiSeq instrument was used for sequencing. Alignment was performed with Burrows-Wheeler Aligner [15] and resulting BAM files were viewed with the Integrative Genomics Viewer [16].

### Sanger sequencing

DNA extracted from tumours was also subject to Sanger sequencing for a *PALB2* variant identified in the corresponding blood DNA according to standard protocols (see Supplementary material).

### Variant description

Variants in this article are described based on transcripts NM_004168.4 for *SDHA* and NM_024675.3 for *PALB2*.

## Results

**Fig. 1:**
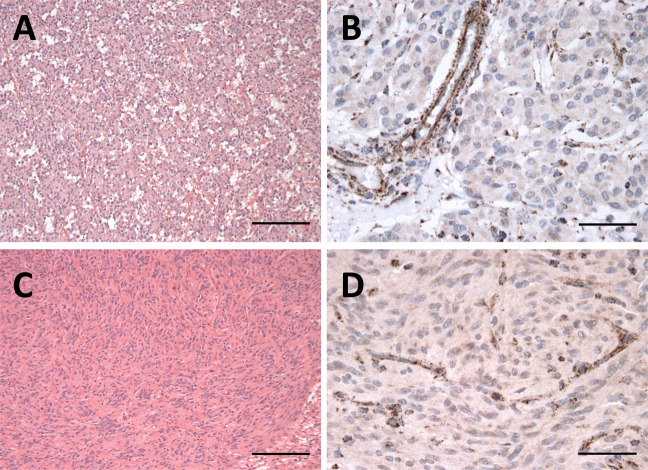
Histology and *SDHB* immunohistochemistry on GIST samples from *SDHA*/*PALB2* diad. **A**, **C** Haematoxylin and eosin staining on samples from son and mother, respectively. **B**, **D** Loss of SDHB immunostaining on samples from son and mother, respectively. Non neoplastic cells show a retained mitochondrial staining pattern which can be appreciated in **B** and **D**.

A 66-year-old female presented with symptoms of satiety and was endoscopically investigated, revealing a 35 mm gastric GIST. She went on to undergo a distal gastrectomy, at which time she was identified as having liver metastases. Further treatment was with imatinib. Subsequently, her son also developed a gastric GIST at the age of 34 years, which was detected as an incidental finding following imaging performed due to trauma. The 53 mm tumour was removed laparoscopically. Histology and immunohistochemistry of both GISTs showed a mixed epithelioid growth pattern and loss of SDHB staining on immunohistochemistry, indicating deficiency of a component of the succinate dehydrogenase (SDH) multiprotein complex (Fig. 1). There was no other reported family history of neoplasia and the proband’s other son had not been diagnosed with any tumours.

The family was referred for Clinical Genetic assessment and initial routine analysis of *SDHB*, *SDHC*, *SDHD*, *KIT* and *PDGFRA* genes in the son’s sample did not reveal a causative constitutional variant and both individuals were recruited to research studies to try and elicit the cause. At the time of investigation (2014), analysis for *SDHA* variants or *SDHC* epimutations was not included in the diagnostic workflow. Following recruitment, the mother was also diagnosed with ductal carcinoma in situ of the breast at the age of 70 years.

WGS on DNA extracted from blood was performed on a research basis. Variant filtering based on a list of 83 cancer predisposition genes and variant assessment according to American College of Medical Genetics criteria revealed a nonsense variant in *SDHA* (c.1534C>T (p.Arg512*)) in both individuals. This variant has previously been reported as associated with GISTs and paragangliomas in the germline heterozygous state [11, 17] and has four submissions in ClinVar [18] with pathogenic or likely pathogenic assertion (two with paraganglioma as the condition for which the assertion is made, one with hereditary cancer predisposition, and one without a condition). Overall allele frequency in gnomAD [19] is 4.9 × 10^−5^ (seven occurrences) with a maximum allele frequency of 9.3 × 10^−5^ in non-Finnish Europeans.

Variant filtering and assessment also identified *PALB2* c.3113 G>A (p.Trp1038*) in blood from both participants. *PALB2* is a breast cancer predisposition gene and this is a frequently identified variant with >10 ClinVar entries with pathogenic assertion, mostly with reference to breast cancer predisposition. Overall allele frequency in gnomAD is 2.1 × 10^−5^ (two occurrences) with a maximum allele frequency of 3.1 × 10^−5^ in non-Finnish Europeans.

Loss of heterozygosity (LOH) analysis was performed on DNA from both tumours to support the role of the *SDHA* variant being causal and also investigate whether there was any evidence for the *PALB2* variant contributing to tumourigenesis.

**Fig. 2:**
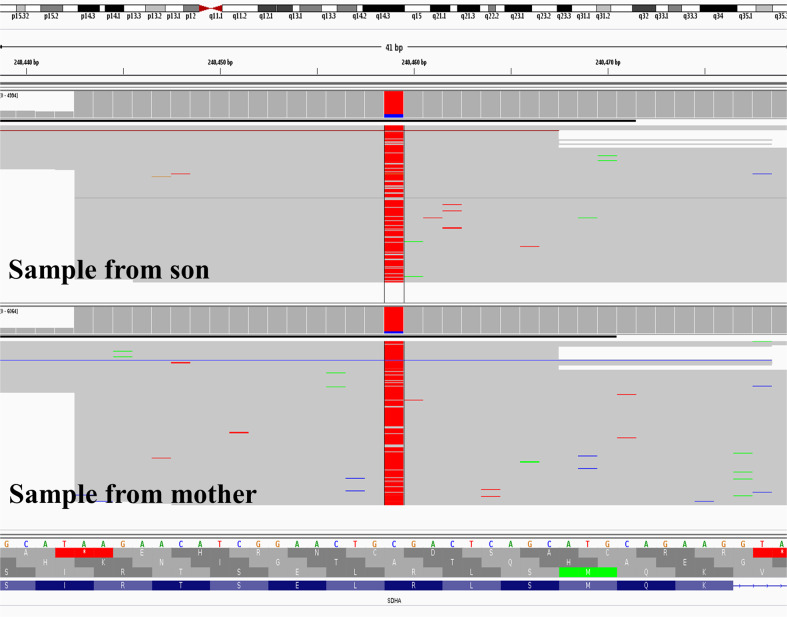
Integrative genomics viewer screenshot from coordinate of *SDHA* variant. BAM files resulting from sequencing DNA extracted from tumour. Loss of *SDHA* wild-type allele shown in samples from both members of diad as indicated by predominance of variant (denoted by red colour) bases.

**Fig. 3:**
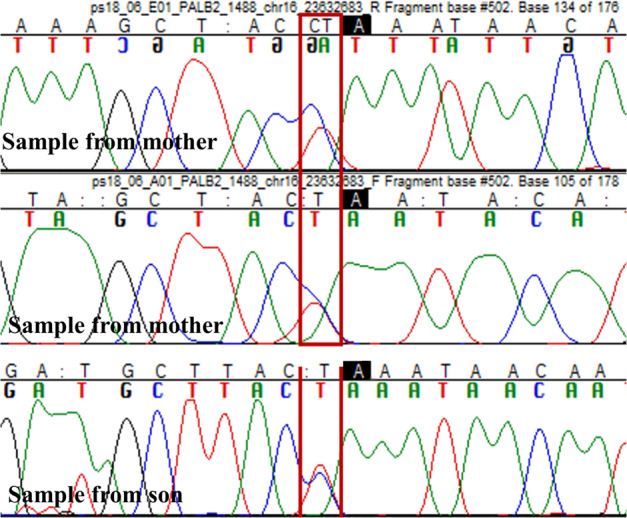
Sanger sequencing chromatograms from coordinate of *PALB2* variant resulting from sequencing DNA extracted from tumour. Retention of wild-type allele indicated by dual peak at site of variant.

Loss of the *SDHA* wild-type allele was confirmed with a panel-based sequencing assay (see Materials (subjects) and methods section) where variant allele fraction (VAF) was 0.42 in the blood sample from the mother and 0.92 in her tumour sample. The son’s samples also showed some evidence of LOH with VAF’s of 0.57 in blood and 0.85 in tumour (Fig. 2). Loss of the wild-type *PALB2* allele, which may have indicated a contribution to increased penetrance of the *SDHA* variant and occurrence in two family members, was not observed (Fig. 3) in either individual using Sanger sequencing

## Discussion

We report a rare case of familial dSDH-wtGIST that was associated with germline-predicted truncating variants in both *SDHA* and *PALB2*.

*SDHA* is a tumour suppressor gene and a second hit through loss of the wild-type allele can be observed in GISTs from germline variant carriers [3]. It encodes a catalytic subunit of the succinate dehydrogenase complex also comprised of components encoded by *SDHB*, *SDHC* and *SDHD*. The succinate dehydrogenase complex locates to the inner mitochondrial membrane and participates in the citric acid cycle by converting succinate to fumarate. Reduced function leads to accumulation of intracellular succinate and resultant inhibition of alpha-ketoglutarate-dependent dioxygenase enzymes including prolyl hydroxylase (PHD). PHD normally promotes hypoxia-inducible-factor 1-alpha (HIF1) breakdown through hydroxylation and reduction in this function leads to a pseudo-hypoxic state with associated upregulation of a range of genes that can promote neoplasia (e.g. IGF-1 and VEGF). Succinate accumulation also leads to oncogenic aberrant methylation via inhibition of TET enzymes [20] and has previously been observed in GISTs [21]. Alternative mechanisms to explain SDH deficiency contributing to tumourigenesis include altered amino acid metabolism and increased reactive oxygen species level as a result of mitochondrial dysfunction [5, 22].

Germline variants in *SDHA*, like other *SDHX* genes, can predispose to the development of PPGL but penetrance for these tumours is estimated to be under 10% from studies of relatives of probands diagnosed with PPGL and analysis of population databases [23–25]. Penetrance for GIST is not well defined but also appears to be low. Studies of 30 *SDHA* variant carriers ascertained by genetic testing for PPGL [23] and 95 carriers with PPGL identified in a literature review [24] each showed one GIST occurring in a family member of a case. Two GISTs were observed amongst ten *SDHA* germline variant carriers found through agnostic cancer genetic testing [26] and four GISTs occurred in 15 carriers identified through a review of UK genetic testing laboratory reports [27].

*PALB2* encodes a protein that interacts with BRCA2 to execute double-stranded DNA repair through homologous recombination (HR) and was identified as a breast cancer predisposition gene in 2007 [28]. Penetrance by age 80 years for (female) breast cancer is currently estimated at 53% whilst the figures for ovarian and pancreatic cancers are 5% and 2–3%, respectively [29]. *PALB2* is widely expressed at RNA and protein level, including in the gastrointestinal tract [30] but no association has previously been made with wtGIST.

Constitutional biallelic variants in *PALB2* can cause Fanconi Anaemia associated with DNA repair deficiency. In individuals with monoallelic variants, a ‘second hit’ at the cellular level can lead to HR DNA repair deficiency and increased repair through the error-prone alternative mechanism of non-homologous end joining (NHEJ) [31]. These changes can be observed by analysis of tumour sequencing data including mutational signatures [32]. One signature is associated with biallelic inactivation of *BRCA1* and *BRCA2* (due to HR deficiency) but has also been demonstrated in breast [33] and pancreatic [34] cancers from individuals with constitutional *PALB2* truncating variants. Around two-thirds of breast cancers from carriers of function compromising *PALB2* variants have a second hit observed as either loss of the wild-type allele or a second somatic variant. Such biallelic deficits correlate closely with evidence of HR deficiency in tumours but tumours without an observable second hit can also occasionally have observable deficiency [31, 35], suggesting a second hit by an unidentified mechanism.

Given that familial wtGIST is rare, we performed further tumour studies to investigate whether there was evidence for the *PALB2* variant contributing to increased penetrance as opposed to being an incidental finding. Tumour studies in both affected individuals supported the primary tumourigenic role of the *SDHA* variant with histology typical of SDH deficiency, loss of SDHB immunostaining (which indicates loss of a SDH complex component and is an indirect measure of SDHA loss) and evidence for loss of the wild-type *SDHA* allele on somatic sequencing. LOH analysis for *PALB2* did not show evidence for loss of the wild-type allele. The *PALB2* variant therefore, may have contributed to the breast cancer occurring in the mother (tumour tissue was unavailable for further analysis) but not the wtGISTs in this family. Preservation of the wild-type *PALB2* allele in tumour studies supports that notion. However, absence of LOH in the context of a tumour suppressor gene does not always imply absent contribution as alternative mechanisms may disrupt the wild-type allele such as epimutation, structural variation not resulting in copy number loss, or a missense variant not assessed as contributory to tumourigenesis. More extensive tumour studies could potentially have been revealing in delineating the contribution of the *PALB2* variant in these GISTs. IHC was useful to demonstrate the role of the *SDHA* variant but no equivalent assay was available for *PALB2*. Whole-genome sequencing was not performed due to inadequate DNA from FFPE tissue but may have revealed evidence of the HR deficient mutational signature that is characteristic of *PALB2* related cancers. Interestingly, SDH deficiency has also been reported to suppress DNA repair by HR [36] so that signature could feasibly be observed in this scenario due to deficiency of either gene or enhanced by the presence of both.

To our knowledge, this is the first case of *SDHA* associated MINAS. The term describes the scenario in which an individual harbours clinically significant germline variants affecting more than one cancer predisposition gene and was suggested to facilitate information sharing about such cases [37]. This occurrence is often reported in the literature but the phenotypic effect is usually unclear, largely due to individual gene combinations being infrequently observed.

A key question following identification of a MINAS case is the nature of the resultant phenotype. Feasible effects include synergy between the variants to produce a more severe or penetrant phenotype, an independent manifestation where each variant leads to cancer risks equivalent to a scenario where the other is not present, and a protective effect due to synthetic lethality. We previously reviewed MINAS cases reported in the literature and generally observed evidence for an independent mechanism (including for the most frequent *BRCA1*/*BRCA2* variant combination) but some cases show severe manifestations (early onset and/or atypical tumours) and cases with a protective effect are less likely to be investigated or reported.

Our studies suggest an independent action of the two variants where the *PALB2* was an incidental finding though further studies would be required to confirm this. Regardless of any contribution to the wtGISTs, the situation produces challenges for genetic counselling in the sense that predictive testing and surveillance must be considered for two variants in the family. Individuals may have different perspectives regarding which of the variants to be tested for and there are likely to be uncertainties as to possible interactive effects between the variants that are difficult to delineate. These scenarios are becoming more frequent as broader genetic testing is applied in clinical settings and to alleviate these uncertainties through collation of cases, we encourage the reporting via the Leiden Open Variant Database (phenotypic tag MINAS) at https://databases.lovd.nl/shared/diseases/04296.

## Supplementary information

Supplemental Material

Consortium group
