# Supplementary material for: Familial wild-type gastrointestinal stromal tumour in association with germline truncating variants in both SDHA and PALB2
Source: Eur J Hum Genet. 2021 Apr 15;29(7):1139–45. doi: 10.1038/s41431-021-00862-5 (PMC8298530; doi:10.1038/s41431-021-00862-5)
Supplement: Supplementary file 1 — Supplemental Material [file 41431_2021_862_MOESM1_ESM.pdf]

Supplementary information - Methods

SDHB immunohistochemistry

SDHB immunohistochemistry was performed on 3-μm sections of FFPE tissue mounted on adhesive slides. The staining was performed on a fully automated system (BOND III IHC and ISH stainer from Leica Biosystems, Nassloch, Germany). The SDHB primary antibody rabbit polyclonal (HPA002868, Sigma Aldrich, St Louis, MO, USA) was used at a dilution of 1:300 after heat-induced epitope retrieval at pH 9.0 (BOND Epitope Retrieval Solution 2, AR9640, Leica Biosystems, Nassloch, Germany) for 20 minutes. The Primary antibody binding to tissue sections was visualized using BOND Polymer Refine Detection system (DS9800, Leica Biosystems, Nassloch, Germany).

Whole genome sequencing (WGS)

WGS and bioinformatic processing to produce variant call format (VCF) files was performed on samples from study participants as part of, and according to protocols devised by, the NIHR BioResource Rare Diseases study.

DNA samples were checked for adequate concentration (30 ng/μl in 110 μl) with the PicoGreen assay (ThermoFisher, Waltham, MA, USA) and DNA degradation with gel electrophoresis. Purity was checked (adequate measurement optical density 260/280 1.75-2.04) with a Trinean DropQuant system (Trinean, Pleasanton, CA, USA). Samples passing quality control checks were shipped on dry ice to the sequencing provider (Illumina Inc., Great Chesterford, UK). Further quality controls were performed by the sequencing provider with a further check for adequate DNA concentration (30 ng/μl) and use of a microarray assay to ensure that samples were able to generate high quality genotyping results (Illumina Infinium Human Core Exome microarray).

0.5μg of the DNA sample was fragmented using Covaris LE220 (Covaris Inc., Woburn, MA, USA) to obtain an average size of 450 base pair (bp) DNA fragments. DNA samples were processed using the Illumina TruSeq DNA PCR-Free Sample Preparation kit (Figure 2.2, Illumina Inc., San Diego, CA, USA) on the Hamilton Microlab Star (Hamilton Robotics, Inc., Reno, NV, USA). The final libraries were checked using the Roche LightCycler 480 II (Roche Diagnostics Corporation, Indianapolis, IN, USA) with KAPA Library Quantification Kit (Kapa Biosystems, Inc., Wilmington, MA, USA) for concentration.

Libraries were sequenced with an Illumina HiSeq 2500 instrument. Files containing sequencing data were delivered to and stored by the University of Cambridge High Performance Computing Service. FASTQ files were generated by HiSeq Analysis Software v2.0 (Illumina Inc., San Diego, CA, USA). Read alignment to GRCh37 was performed using Illumina Isaac aligner version SAAC00776.15.01.27.(1) Single nucleotide variants and indels were called from resulting binary compressed sequence alignment map (BAM) files using Illumina Starling software version 2.1.4.2.

Annotation of variants was performed according to the downstream analysis used in this project (see relevant chapters) but frequently utilised UK10K(2) allele frequency information that was added to variants by BRIDGE annotation pipelines.

Variant assessment from WGS data

Variants occurring in genes included in a list of 83 cancer predisposition genes were filtered and assessed according to a protocol described previously.(3)

DNA extraction from formalin fixed paraffin embedded tumour blocks

Slides were prepared from formalin fixed paraffin embedded (FFPE) tumour blocks by the Human Research Tissue Bank, Cambridge University Hospitals. De-paraffinisation was

performed by soaking in 100% xylene, 100% ethanol and air drying. In order to optimise the amount of tumour material contributing to sequencing results, slides were reviewed by a pathologist to mark selected tissue and tumour dissection was performed by colleagues in the Department of Haematology and Oncology diagnostic services, Cambridge University Hospitals. Resulting tissue was placed in ATL tissue lysis buffer (Qiagen, Hilden, Germany) with proteinase K added before incubation. DNA was purified from the resulting lysate with a QiaAmp MinElute Column (Qiagen, Hilden, Germany).

Ampliseq panel sequencing

Library preparation was undertaken by the colleagues in the Stratified Medicine Core Laboratory using a custom Ampliseq panel (Thermo Fisher Scientific, Waltham, MA, USA) that included the *SDHA* region of interest. The protocol was adapted from a NEBNext Ultra II protocol for Illumina sequencing (New England Biolabs Inc., Ipswich, MA, USA). DNA samples were made up 10ng in 5µl and transferred to a 96 well plate with two primer pools (to avoid competition for hybridisation between adjacent primer pairs). Consequently, two wells were used per sample. Polymerase chain reactions (PCR) were performed by adding Q5 mastermix (New England Biolabs Inc., Ipswich, MA, USA) (Table S1) to each well and thermal cycling under the protocol described in Table S2. Following completion of PCR reactions, adaptor sequences were removed from amplicons by the addition of NEB USER Enzyme (New England Biolabs, Ipswich, MA, USA), which cleaves nucleic acids at uracil bases, and incubation with a thermal cycler. Wells corresponding to each sample for both primer pools were combined and transferred to wells of a MIDI plate containing 1.8X Agencourt AMpure XP magnetic beads (Beckman Coulter, Pasadena, CA, USA) to bind to amplicons. Two rounds of pull down and re-suspension were undertaken. To ligate specific barcode sequences to amplicons from specific samples, NEB End Repair reaction buffer then NEB End Repair enzyme mix (New England Biolabs Inc., Ipswich, MA, USA) was added to each well. 30µl NEB ligation master mix, 1µl of ligation enhancer and 2µl barcode sequence solution was added to each well before mixing and incubation. Further clean up using AMpure beads with ethanol washes were carried out. Quality of prepared libraries was measured by subjecting a 1/1000 dilution of each sample to quantitative PCR according to a KAPA protocol (Illumina Inc., San Diego, CA, USA). A 5µl aliquot of each sample was then transferred to an Illumina MiSeq instrument for sequencing. Alignment was performed with Burrows Wheeler Aligner(4) and resulting BAM files were viewed with the Integrative Genomics Viewer.(5)

Table S1 - PCR reaction components for Ampliseq panel

Reaction component	Volume (µl)
Q5 Master Mix	25
Primer Mix	10
DNA	5
Water	10
Total volume	50

Table S2 - PCR thermal cycling protocol for Ampliseq panel – 30 cycles

Step	Temperature (°C)	Duration (secs)
Initial denaturation	98	30
Denature	98	10
Anneal	60	30

Extend	65	120
Final extension	65	300
Hold	4	Hold

Sanger sequencing
DNA extracted from tumours was also subject to Sanger sequencing for a *PALB2* variant identified in the corresponding blood DNA, performed by colleagues in the Department of Medical Genetics, University of Cambridge. PCR reactions for the region of interest were undertaken according to the advised protocol for AmpliTaq Gold DNA polymerase 50µl reaction (Thermo Fisher Scientific, Waltham, MA, USA). Primers are shown in Table S3 and reaction constituents are described in Table S4. Thermal cycling was performed on a Tetrad PTC-225 (MJ research, Waltham, MA, USA) according to the protocol described in Table S5. PCR products were subject to gel electrophoresis in 1% agarose gel (90v/40mins) and photographed under ultraviolet light to check for an observable band of predicted length. Following PCR, excess primers and deoxynucleotides were removed by adding a mixture of Exonuclease I (New England Biolabs, Ipswich, MA, USA) and Shrimp Alkaline Phosphatase (GE Healthcare, Chicago, IL, USA) to each PCR product well and incubating. Bidirectional Sanger sequencing of resulting products was performed with BigDye Terminator Version 3.1 Cycle Sequencing Kit (Applied Biosystems, Foster City, CA, USA) according to the reaction constituents described in Table S6 and thermal cycling protocol (with a Tetrad PTC-225) outlined in Table S7. To remove unincorporated dye, 40µl of 75% isopropanol was added to each well after the sequencing reaction. The plate containing the wells was then centrifuged and inverted onto absorbent paper to remove supernatant. It was left to air dry in dark conditions before adding 10µl of Hi-Di™ Formamide (Applied Biosystems, Foster City, CA, USA) to each well. The plate was then placed on an ABI 3131xl sequence analyser (Applied Biosystems, Foster City, CA, USA). Resulting chromatogram files were analysed with Sequencher 5.3 software (Gene Codes Corporation, Ann Arbor, MI, USA).

Table S3 - Primers used for amplifying region containing *PALB2* variant

Forward primer	CAACAGCAACACAAAACCACA
Reverse primer	AACTTTTGCTGAGGTCCAAGG

Table S4 - PCR reaction components for Sanger sequencing

**Reaction component**	**Volume (µl)**
AmpliTaq Gold DNA polymerase (5U/µl)	0.25
10µm Primer Mix	2
DNA	5
Water	33.75
10nM dNTP mix	1
25nM $MgCl_2$	3
10X PCR buffer	5
Total volume	50

Table S5 - PCR thermal cycling protocol for Sanger sequencing – 32 cycles

**Step**	**Temperature (°C)**	**Duration**
Initial denaturation	95	10 mins
Denature	95	15 secs

Anneal	59	30 secs
Extend	72	1 minute per kb
Final extension	72	5 mins
Hold	4	Indefinite

Table S6 - Sanger sequencing reaction components

Reaction component	Volume (µl)
BigDye Terminator Version 3.1	0.75
Primer solution (10pmol)	1
5x BigDye sequencing buffer	2
Water	4.25

Table S7 - Thermal cycling protocol for Sanger sequencing reaction – 20 cycles

Step	Temperature (°C)	Duration (secs)
Denature	96	10
Anneal	50	5
Extend	60	210

References

1. Raczy C, Petrovski R, Saunders CT, Chorny I, Kruglyak S, Margulies EH, et al. Isaac: Ultra-fast whole-genome secondary analysis on Illumina sequencing platforms. Bioinformatics. 2013;29(16):2041–3.
2. Walter K, Min JL, Huang J, Crooks L, Memari Y, McCarthy S, et al. The UK10K project identifies rare variants in health and disease. Nature. 2015;526(7571):82–9.
3. Whitworth J, Smith PS, Martin JE, West H, Luchetti A, Rodger F, et al. Comprehensive Cancer-Predisposition Gene Testing in an Adult Multiple Primary Tumor Series Shows a Broad Range of Deleterious Variants and Atypical Tumor Phenotypes. Am J Hum Genet. 2018;103(1):3–18.
4. Li H, Durbin R. Fast and accurate short read alignment with Burrows-Wheeler transform. Bioinformatics. 2009;25(14):1754–60.
5. Robinson JT, Thorvaldsdóttir H, Winckler W, Guttman M, Lander ES, Getz G, et al. Integrative genomics viewer. Nat Biotechnol. 2011;29(1):24–6.
